# Oral azithromycin given during labour decreases bacterial carriage in the mothers and their offspring: a double-blind randomized trial

**DOI:** 10.1016/j.cmi.2016.03.005

**Published:** 2016-06

**Authors:** A. Roca, C. Oluwalana, A. Bojang, B. Camara, B. Kampmann, R. Bailey, A. Demba, C. Bottomley, U. D'Alessandro

**Affiliations:** 1) Medical Research Council Unit The Gambia; 2) London School of Hygiene and Tropical Medicine, London, UK; 3) Ministry of Health and Social Welfare, Gambia; 4) Institute of Tropical Medicine, Antwerp, Belgium

**Keywords:** Azithromycin, bacterial carriage, neonatal sepsis, randomized clinical trial, sub-Saharan Africa

## Abstract

Bacterial sepsis remains a leading cause of death among neonates with *Staphylococcus aureus*, group B streptococcus (GBS) and *Streptococcus pneumoniae* identified as the most common causative pathogens in Africa. Asymptomatic bacterial colonization is an intermediate step towards sepsis. We conducted a phase III, double-blind, placebo-controlled randomized trial to determine the impact of giving one oral dose of azithromycin to Gambian women in labour on the nasopharyngeal carriage of *S. aureus*, GBS or *S. pneumoniae* in the newborn at day 6 postpartum. Study participants were recruited in a health facility in western Gambia. They were followed for 8 weeks and samples were collected during the first 4 weeks. Between April 2013 and April 2014 we recruited 829 women who delivered 843 babies, including 13 stillbirths. Sixteen babies died during the follow-up period. No maternal deaths were observed. No serious adverse events related to the intervention were reported. According to the intent-to-treat analysis, prevalence of nasopharyngeal carriage of the bacteria of interest in the newborns at day 6 was lower in the intervention arm (28.3% versus 65.1% prevalence ratio 0.43; 95% CI 0.36–0.52, p <0.001). At the same time-point, prevalence of any bacteria in the mother was also lower in the azithromycin group (nasopharynx, 9.3% versus 40.0%, p <0.001; breast milk, 7.9% versus 21.6%, p <0.001; and the vaginal tract, 13.2% versus 24.2%, p <0.001). Differences between arms lasted for at least 4 weeks. Oral azithromycin given to women in labour decreased the carriage of bacteria of interest in mothers and newborns and may lower the risk of neonatal sepsis.

**Trial registration**ClinicalTrials.gov Identifier NCT01800942.

## Background

There are more than 4 million neonatal deaths annually and a third are caused by severe bacterial disease, which mainly presents as sepsis [1]. In sub-Saharan Africa, the limited available data show that neonatal sepsis is caused by both Gram-positive and Gram-negative bacteria [2], [3], [4], although more than half of the cases are attributable to the former, particularly *Staphylococcus aureus* [1], *Streptococcus pneumoniae* and group B streptococcus (GBS) [3].

Early-neonatal sepsis is mainly due to intrapartum bacterial vertical transmission [2] during delivery (in the birth canal) or during the first weeks of life as a result of the close physical contact with the mother if she carries pathogenic bacteria [1], [5], [6], [7]. If newborns are mainly infected by their mother, an intervention that is able to reduce maternal bacterial carriage should prevent vertical transmission and consequently neonatal sepsis.

Azithromycin is a macrolide with a wide antimicrobial spectrum [8] currently licensed for use in children >6 months of age for a wide range of infections [9], [10]. As part of the WHO-recommended trachoma control strategy, mass azithromycin treatment campaigns in countries where trachoma is endemic [11], [12], [13] decreased both the nasopharyngeal pneumococcal carriage [14] and the overall childhood mortality [15].

Azithromycin has also been used in pregnant women in sub-Saharan Africa in several trials designed to reduce the incidence of maternal malaria and preterm deliveries and low-birthweight, but a meta-analysis found no effect of these outcomes [16]. However, a recent study conducted in Papua New Guinea showed 25% reduction of low-birthweight infants after including monthly azithromycin (4 g) for 3 months to the standard sulphadoxine-pyrimethamine during the last months of pregnancy [17]. Blocking vertical transmission has already been used successfully in the context of GBS, the main bacterium causing neonatal sepsis in developed countries, in Europe [18] and USA [19]. But whereas in Europe and the USA treatment is targeted at women with GBS vaginal carriage, in sub-Saharan Africa systematic treatment may be more feasible, since half of pregnant women are carriers of bacteria associated with neonatal sepsis in the region [20]. In a first proof-of-concept assessing the potential of a new intervention to prevent neonatal sepsis, we evaluated the efficacy of one oral dose of azithromycin administered to women in labour in decreasing bacterial carriage (*S. aureus*, GBS and *S. pneumoniae*) both in the mother and her newborn.

### Methods/Design

The study protocol has been published elsewhere [21]. Briefly, this was a phase III, double-blind, placebo-controlled, randomized trial in which women in labour were randomized to receive a single dose of oral azithromycin (2 g) or placebo.

The packaging and labelling of the interventional medical product was conducted by IDIFARMA. Azithromycin and placebo were provided as tablets packed in blisters. IDIFARMA created the randomization list (permuted blocks) and numbered the blisters according to the list. One blister pack of interventional medical product contained four tablets each of 0.5 g of azithromycin or placebo (2 g). The active drug and the placebo looked identical. The statistician of the Data Safety Monitor Board (DSMB) kept the list until the final database was locked. The investigators were blinded to the patient's allocation until the database was locked, when the code was broken.

The study was based at the Jammeh Foundation for Peace (JFP), a government-run health centre located in western Gambia that manages 4500 deliveries/year. The population in the catchment area is representative of The Gambia and it covers the main ethnic groups. Approximately 70% of deliveries in the country occur in health facilities (Jasseh *et al*. personal communication). The climate of the area is typical of the sub-Sahel region. Illiteracy is high [22].

Between April 2013 and April 2014, women in labour aged 18–45 years were recruited when attending the JFP labour ward. They had signed consent to participate in the study during their antenatal visits. Eligibility was re-assessed in the JFP labour ward based on the exclusion criteria: (i) known human immunodeficiency virus infection; (ii) any acute or chronic condition that could interfere with the study as judged by the research clinicians; (iii) planned travel out of the catchment during the follow up; (iv) known risk of caesarean section; (v) likely referral during labour (eclampsia or severe anaemia); (vi) known multiple pregnancy; (vii) known severe congenital malformation or intrauterine death confirmed before randomization; (ix) known allergy to macrolides; (x) consumption of antibiotic within the previous week.

Pre-intervention samples were collected during labour (nasopharyngeal swab (NPS) and vaginal swab (VS)). An NPS was collected from the baby within 6 h after birth. After discharge, mothers and babies were visited at home for 2 months, daily during the first week and weekly thereafter. NPS and breast milk samples were collected at days 3, 6, 14 and 28. In addition, a VS was collected between days 8 and 10 after delivery at the postnatal check visit at JFP.

The primary end point was prevalence of carriage of *S. aureus*, GBS or *S. pneumoniae* in the NPS sample of the newborn at day 6. Secondary end points included: (i) bacterial carriage in the NPS of the baby and the mother; (ii) carriage in the VS and breast milk during the first 4 weeks after delivery; and (iii) prevalence of carriage of any of the study bacteria non-susceptible to azithromycin.

To evaluate the safety of the intervention on mothers and newborns, adverse events were monitored and assessed throughout the follow up. Diagnoses were based on clinical judgement according to the study clinicians.

A local safety monitor (LSM) and a DSMB reviewed serious adverse events during the trial, and the trial was monitored by an independent clinical trials monitor. The study was approved by the joint Gambia Government/Medical Research Council (MRC)/Ethics Committee.

### Sample collection and laboratory analysis

The NPS, low VS and breast milk samples were collected as part of the trial; see details elsewhere [24].

### Laboratory procedures

Samples were processed following standard microbiological procedures [21].

### Sample size rationale

The sample size was chosen to provide 88% power to detect a 20% reduction in the primary end point (i.e. from 60% [23] to 48%). We assumed that NPS would not be available for 15% of newborns at day 6.

### Data management and statistical analysis

Case-report-forms and laboratory forms were reviewed before being double entered into *OpenClinica* (www.openclinica.com).

The analysis was carried out using Stata (version 13.1). We compared the prevalence of bacterial carriage in mothers and newborns allocated to the azithromycin and placebo groups (i.e. intent-to-treat analysis). Additional analyses included carriage acquisition rates for the periods 0–6 days and 7–28 days. Ratios and 95% CI were calculated for each comparison, and Fisher's exact test was used to compute p values. We included twins, but did not adjust the 95% CI and significance tests for the effect of clustering because the design effect was negligible.

A Poisson regression model was used to assess the effect of the intervention on prevalence of bacterial carriage across all time-points. The model included the baseline carriage status of the mother and time as covariates, and robust standard errors were used to account for the dependence between observations from the same individual as well as the model misspecification (carriage status does not follow a Poisson distribution).

For the primary end point, three additional analyses were performed: (i) a sub-group analysis in women who delivered 2 hours or more after taking the drug; (ii) a sensitivity analysis using multiple imputation in which bacterial carriage was imputed from baseline demographic data (age and ethnicity for mothers, and sex and birthweight for newborns) and from carriage data at other time-points; and (iii) a per protocol analysis.

## Results

### Study population

In all, 1061 women in labour were assessed for eligibility and 829 (78.1%) were recruited, randomized and treated (414 azithromycin and 415 placebo) (see Table 1).

**Fig. 1:**
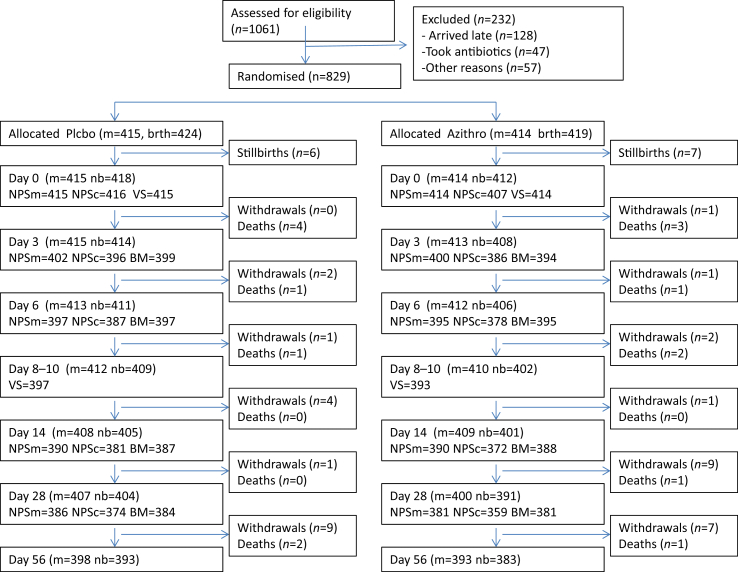
Trial profile. Abbreviations: nb, Newborns; m, Mothers; NPSn, nasopharyngeal swabs collected from newborns; NPSm, nasopharyngeal swabs collected from mothers; BM, breast milk samples collected; VS, vaginal swabs collected. Some participants were still present until follow up but their samples were missed in some of the visits.

These 829 recruited women delivered 843 babies (Fig. 1). Seven neonatal deaths (4.7%) had a clinical diagnosis of sepsis, meningitis or pneumonia according to the adverse events form; four in the placebo group (none with underlying conditions), and three in the azithromycin group (all with underlying conditions—one each of congenital heart disease, suspicion of vitamin K deficiency with haemorrhagic disease, and aspiration of gastric content). There were no maternal deaths reported. Of the 25 maternal hospitalizations, 18 (72%) were due to labour complications and three had diagnosis of puerperal sepsis (one in the azithromycin group and two in the placebo group). No adverse events/serious adverse events related to the intervention were reported for the newborns. One mother developed moderate urticarial rash that lasted for 3 days.

### Prevalence of carriage

**Fig. 2:**
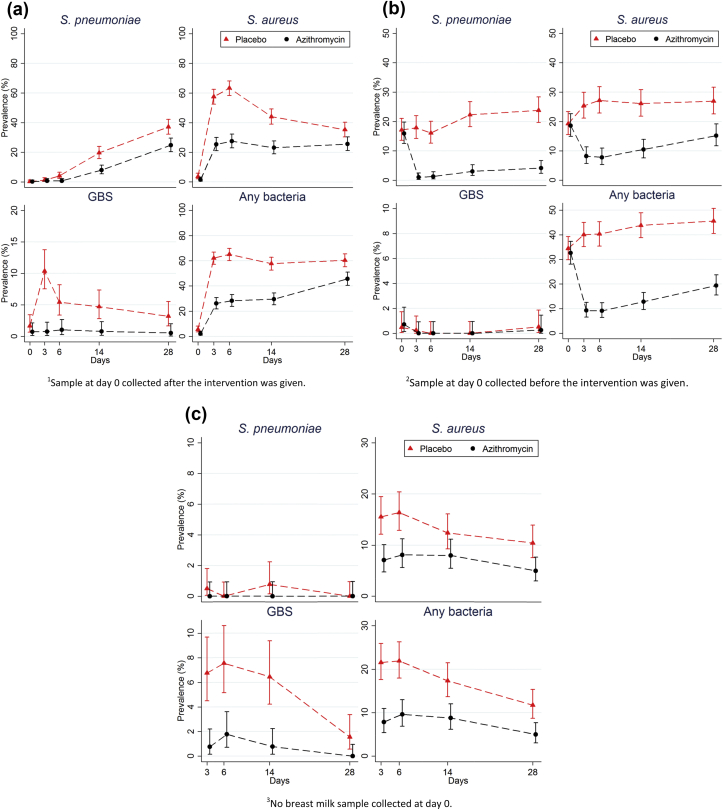
Bacterial carriage of *Staphylococcus aureus*, group B streptococci (GBS) and *Streptococcus pneumoniae* in the different study time points for the azithromycin (AZI) and placebo groups. (a) Newborn nasopharyngeal swabs (NPS); (b) Maternal NPS; (c) Breast milk sample.

At day 6, samples were collected from 93% of the newborns that were alive and at all other times at least 90% of mothers and newborns provided samples (Table 2, Fig. 2).

*Pre-intervention samples*. Prevalence of carriage in VS and NPS was similar between study arms for all bacteria.

*Post-intervention NPS*. All study bacteria were less common in newborns in the azithromycin group than in the placebo group. At day 6, the prevalence of nasopharyngeal carriage of study bacteria in newborns was 28.3% in the azithromycin group versus 65.1% in the placebo group (prevalence ratio (PR) = 0.43; p <0.001). Similarly, in mothers bacterial carriage was lower in the azithromycin group, with PR ranging from 0.08 (for *S. pneumoniae* at day 6) to 0.57 (for *S. aureus* at day 28).

*Post-intervention breast milk samples*. The prevalence of carriage of study bacteria in breast milk was significantly lower among mothers from the azithromycin group at day 6 (9.6% versus 21.9%; PR = 0.44; p <0.004).

*Post-intervention VS*. The prevalence of carriage of study bacteria in the VS collected post-intervention was lower in the azithromycin group than in the placebo group (13.2% versus 24.2%; PR = 0.55; p <0.001). *S. pneumoniae* was not isolated from VS.

*Antibiotic resistance*. Isolates resistant to azithromycin, particularly *S. aureus*, were more common in the intervention group for all sample types (Table 3).

*Sensitivity analyses*. Differences in bacterial carriage between trial arms in the analysis stratified by time of treatment (see Supplementary material, Table S1), the per protocol analysis (see Supplementary material, Table S2) and the analysis of carriage acquisition (see Supplementary material, Table S3) were similar to those observed in the primary analysis. For mothers recruited <2 h before delivery, prevalence of nasopharyngeal carriage of study bacteria at day 6 in their newborns was 35.5% if they received azithromycin and 67.5% if they received placebo (PR = 0.53; p <0.001).

### Use of antibiotic during the follow-up period

The intervention reduced antibiotic prescription in the study women by 40% (6.0% versus 10.1% for any antibiotic, PR = 0.58, 95% CI 0.36–0.94; p = 0.031), but not in the newborns (10.0% versus 10.1% in the azithromycin and placebo groups, respectively; p 1.000).

## Discussion

One oral dose (2 g) of azithromycin given to women in labour substantially reduced the prevalence of *S. aureus*, GBS and *S. pneumoniae* carriage both in the newborn and the mother. The difference between arms was already evident at birth (before breast feeding), probably as a result of the clearance of bacterial pathogens from the birth canal, and was maintained during the entire neonatal period. The prolonged effect of azithromycin on neonatal bacterial carriage is probably attributable to its substantial effects on carriage in the mother's nasopharynx and breast milk and the presence of azithromycin in the breast milk [24].

Several studies, including one in The Gambia [14], have shown that azithromycin can reduce nasopharyngeal carriage of *S. pneumoniae* when given as prophylaxis but no studies have investigated whether azithromycin has an effect on GBS, and few have investigated whether it has an effect on *S. aureus*, both leading causes of neonatal sepsis in sub-Saharan Africa [3], [4] and the latter the bacterium most commonly isolated from the nasopharynx in newborns [23]. A recent study comparing the impact of sulfadoxine-pyrimethamine in combination with three courses of 4 g of azithromycin versus sulfadoxine-pyrimethamine with chloroquine given monthly during pregnancy found no difference between groups on the prevalence of *S. aureus* in the maternal nasopharynx at delivery [25].

Group B streptococci were almost eliminated in mothers treated with azithromycin and the prevalence was very low in their offspring. This may have implications beyond sub-Saharan Africa as GBS is a common cause of early neonatal sepsis in Europe and the USA where pregnant women are screened for GBS and, if positive, are treated during delivery with intravenous antibiotic [18], [26]. Intravenous prophylactic treatment with penicillin is only recommended if the women attend the maternal ward at an early stage of labour [18]. Our data show that azithromycin can prevent vertical transmission of GBS and the other study bacteria even when taken <2 h before delivery.

The prevalence of azithromycin resistance among bacterial isolates in the intervention arm was high, particularly for *S. aureus.* Although this may be worrying, such resistance is unlikely to be sustained as resistance falls in the absence of antibiotic pressure because of the associated fitness cost [27]. In The Gambia, after mass campaigns of azithromycin treatment, *S. pneumoniae* macrolide resistance returned to baseline levels within 6 months [14]. Still, the selection of resistance after azithromycin treatment should be closely monitored in future studies because if larger studies show an effect on severe clinical outcomes, the intervention proposed would lead to continuous and sustained antimicrobial pressure. The importance in clinical care of such resistance is limited considering that azithromycin is currently not used for clinical care in The Gambia. Furthermore, as we found that the intervention reduced antibiotic use in mothers by >40% during the follow-up period, future larger studies should also monitor any effect on the intervention in reducing resistance to common antibiotics.

The trial was designed as a proof-of-concept, to assess whether azithromycin can reduce bacterial carriage in the mother and in her offspring. It was not powered to assess the intervention's effect on neonatal morbidity or mortality, neither did it attempt to standardize clinical end points. The diagnosis of neonatal sepsis was made on a clinical basis and supported by chest X-rays or laboratory results at admission.

Compared with the general population, study participants benefited from better care, which probably decreased morbidity and mortality in both arms. If a study nurse suspected an infection during a home visit, the participant was immediately referred to the study clinicians who managed them accordingly. Such close follow up may have increased the probability of hospitalization in children with mild signs/symptoms of disease who, in a real-life situation, would not have been taken to a health facility, and this may have contributed to decreasing the possible difference between arms. Although both arms had a comparable number of deaths due to severe infections (sepsis, pneumonia and meningitis), deaths in the intervention arm, unlike the placebo arm, occurred in newborns with an underlying severe condition.

Azithromycin treatment during labour can significantly reduce bacterial carriage among newborns and may therefore lower their risk of neonatal sepsis, pneumonia and meningitis. This simple intervention could therefore have a dramatic impact on neonatal mortality, and might also prevent puerperal sepsis and maternal deaths. A larger randomized controlled clinical trial is urgently needed to determine whether the intervention is effective against these clinical outcomes. Such a trial should also monitor the effect of the intervention on the spread of azithromycin resistance in the community.

## Transparency Declaration

All authors declare no competing interest.

## Author's contributions

AR and UDA conceived the study. AR designed the study, drafted the protocol and wrote the initial manuscript. UDA contributed significantly in the final version of the design, protocol and manuscript. CO, BC and AB developed and adapted the field and laboratory work and made contributions to the development of the manuscript. CB led the statistical analysis plan document, conducted the statistical analysis of the trial and contributed to the manuscript. AD, RB and BK contributed to the study protocol and the manuscript. All authors read and approved the final manuscript.

## Funding

The MRC Unit in The Gambia receives core funding from the MRC UK. This trial was jointly funded by the UK MRC and the UK Department for International Development (DFID) under the MRC/DFID Concordat agreement (reference number MR/J010391/1) and is also part of the EDCTP2 programme supported by the European Union.

## Figures and Tables

**Table 1:** Baseline characteristics of study participants

	Azithromycin	Placebo
**Mothers' characteristics**	**(*n* = 414)**	**(*n* = 415)**
Age, median (interquartile range)	26.0 (22.0–30.0)	25.0 (22.0–30.0)
Ethnicity, *n* (%)		
Madinka	161 (40.1)	187 (45.8)
Fula	77 (19.2)	64 (15.7)
Jola	68 (17.0)	56 (13.7)
Other	95 (23.7)	101 (24.8)
Season of delivery, *n* (%)^a^	141 (34.1)	143 (34.5)
Mode of delivery, *n* (%)		
Vaginal	404 (97.6)	410 (98.8)
Caesarean	10 (2.4)	5 (1.2)
Multiple pregnancy, *n* (%)	5 (1.2)	9 (2.2)
Hours from treatment to delivery, median (interquartile range)	3.2 (1.1,8.3)	2.9 (1.3,6.3)
Hours from rupture of membrane to delivery, median (interquartile range)^b^	0.4 (0.1,1.8)	0.3 (0.1,1.3)
**Newborn's characteristics**	**(*n* = 419)**	**(*n* = 424)**
Gender, females *n* (%)	207 (49.4)	198 (46.7)
Apgar score at birth, *n* (%)		
0	6 (1.4)	6 (1.4)
1–6	8 (1.9)	5 (1.2)
7–10	402 (96.6)	408 (97.4)
Weight, *n* (%)^c^	3.1 (2.8–3.5)	3.1 (2.9–3.4)
Gestational age (weeks), *n* (%)^d^	36.0 (35.0–38.0)	36.0 (35.0–38.0)

a Rainy season: children born June to October.

b Time of rupture of membranes is missing in *n* = 441 (230 in the azithromycin and 211 in the placebo arm).

c Weight missing in *n* = 2 (both in the placebo arm).

d Gestational age missing in *n* = 33 (16 in the azithromycin and 17 in the placebo arm).

**Table 2:** Bacterial carriage in newborns and mothers Intent-to-treat analysis; nasopharyngeal swab and breast milk samples

	Day 0^a^				Day 3				Day 6^b^				Day 14				Day 28			
P'bo (%)	AZI (%)	PR (95% CI)	p value	P'bo (%)	AZI (%)	PR (95% CI)	p value	P'bo (%)	AZI (%)	PR (95% CI)	p value	P'bo (%)	AZI (%)	PR (95% CI)	p value	P'bo (%)	AZI (%)	PR (95% CI)	p value
**NPS newborn**	*n* = 416	*n* = 417			n = 396	n = 386			n = 387	n = 378			n = 381	*n* = 372			*n* = 374	*n* = 359		
GBS	1.7	0.7	0.44 (0.11–1.68)	0.341	10.4	0.8	0.08 (0.02–0.24)	<0.001	5.4	1.1	0.20 (0.07–0.56)	0.001	4.7	0.8	0.17 (0.05–0.57)	0.001	3.2	0.6	0.17 (0.04–0.77)	0.012
*Streptococcus pneumoniae*	0.2	0.2	1.02 (0.06–16.3)	1.000	1.3	0.8	0.62 (0.15–2.56)	0.725	4.1	0.8	0.19 (0.06–0.65)	0.004	19.7	8.1	0.41 (0.28–0.61)	<0.001	37.2	24.8	0.67 (0.53–0.83)	<0.001
*Staphylococcus aureus*	3.6	1.5	0.41 (0.16–1.04)	0.075	57.6	25.4	0.44 (0.36–0.53)	<0.001	63.3	27.5	0.43 (0.36–0.52)	<0.001	44.1	23.1	0.52 (0.42–0.65)	<0.001	35.3	25.6	0.73 (0.58–0.91)	0.005
**Any bacteria**	5.5	2.2	0.40 (0.19–0.85)	0.018	62.1	26.2	0.42 (0.35–0.51)	<0.001	65.1	28.3	0.43 (0.36–0.52)	<0.001	57.7	29.6	0.51 (0.43–0.61)	<0.001	60.4	45.7	0.76 (0.66–0.87)	<0.001

**NPS mother**	*n* = 415	*n* = 414			*n* = 402	*n* = 400			*n* = 397	*n* = 395			*n* = 390	*n* = 390			*n* = 386	*n* = 381		
GBS	0.5	0.7	1.50 (0.25–8.95)	0.686	0.2	0	NA	NA	0	0	NA	NA	0	0	NA	NA	0.5	0.3	0.51 (0.05–5.56)	1
*S. pneumoniae*	17.1	15.9	0.93 (0.69–1.27)	0.709	17.9	1.0	0.06 (0.02–1.15)	<0.001	16.1	1.3	0.08 (0.03–0.19)	<0.001	22.3	3.1	0.14 (0.08–0.25)	<0.001	23.8	4.2	0.18 (0.11–0.29)	<0.001
*S. aureus*	19.3	18.6	0.96 (0.73–1.28)	0.859	25.4	8.3	0.33 (0.23–0.47)	<0.001	27.2	7.8	0.29 (0.20–0.42)	<0.001	26.2	10.5	0.40 (0.29–0.56)	<0.001	26.9	15.2	0.57 (0.42–0.75)	<0.001
**Any bacteria**	34.5	32.6	0.95 (0.78–1.15)	0.607	40.0	9.3	0.23 (0.17–0.32)	<0.001	40.3	9.1	0.23 (0.16–0.32)	<0.001	43.8	12.8	0.29 (0.22–0.39)	<0.001	45.6	19.4	0.43 (0.34–0.54)	<0.001

**Breast milk**	*n* = 0	*n* = 0			*n* = 399	*n* = 394			*n* = 397	*n* = 385			*n* = 387	*n* = 388			*n* = 384	*n* = 381		
GBS	—	—	—	—	6.8	0.8	0.11 (0.33–0.37)	<0.001	7.6	1.8	0.23 (0.10–0.53)	<0.001	6.5	0.8	0.12 (0.04–0.39)	<0.001	1.6	0.0	NA	0.031
*S. pneumoniae*	—	—	—	—	0.5	0	NA	NA	0	0	NA	NA	0.8	0.0	NA	0.124	0.0	0.0	NA	NA
*S. aureus*	—	—	—	—	15.5	7.1	0.46 (0.30–0.70)	<0.001	16.4	8.1	0.49 (0.33–0.74)	<0.001	12.4	8.0	0.64 (0.42–0.99)	0.044	10.4	5.0	0.48 (0.28–0.81)	0.006
**Any bacteria**	—	—	—	—	21.6	7.9	0.37 (0.25–0.54)	<0.001	21.9	9.6	0.44 (0.31–0.63)	<0.001	17.3	8.8	0.51 (0.33–0.75)	<0.001	11.7	5.0	0.43 (0.25–0.71)	0.001

**Vaginal swabs**	*n* = 415	*n* = 414			*n* = 0	*n* = 0			*n* = 396	*n* = 386			*n* = 0	*n* = 0			*n* = 0	*n* = 0		
GBS	15.9	18.1	1.14 (0.84–1.54)	0.407	—	—	—	—	13.6	4.6	0.4 (0.20–0.56)	<0.001	—	—	—	—	—	—	—	—
*S. pneumoniae*	0	0	NA	NA	—	—	—	—	0	0	NA	NA	—	—	—	—	—	—	—	—
*S. aureus*	16.6	12.3	0.74 (0.53–1.04)	0.093	—	—	—	—	14.1	8.9	0.63 (0.42–0.94)	0.026	—	—	—	—	—	—	—	—
**Any bacteria**	28.9	25.1	0.87 (0.69–1.09)	0.241	—	—	—	—	24.2	13.2	0.55 (0.40–0.74)	<0.001	—	—	—	—	—	—	—	—

Abbreviations: AZI, azithromycin; GBS, group B streptococci; NPS, nasopharyngeal swab; P'bo– placebo; PR, Prevalence Ratio.

a At day 0, nasopharyngeal and vaginal swabs in mothers were collected before the intervention and nasopharyngeal swabs in newborns after the intervention.

b Vaginal swabs were collected at day 8–10 instead.

**Table 3:** Prevalence of isolates resistant to azithromycin in newborns and mothers in the different study samples

	Day 0^a^				Day 3				Day 6^b^				Day 14				Day 28			
P'bo (%)	AZI (%)	PR (95% CI)	p value	P'bo (%)	AZI (%)	PR (95% CI)	p value	P'bo (%)	AZI (%)	PR (95% CI)	p value	P'bo (%)	AZI (%)	PR (95% CI)	p value	P'bo (%)	AZI (%)	PR (95% CI)	p value
**NPS newborn**	*n* = 416	*n* = 417			*n* = 396	*n* = 386			*n* = 387	*n* = 378			*n* = 381	*n* = 372			*n* = 374	*n* = 359		
GBS	0	0	NA	NA	0.5	0	NA	NA	0.5	0.3	0.51 (0.05–5.62)	1	0.3	0.3	1.02 (0.06–16.31)	1	0.0	0.3	NA	NA
*Streptococcus pneumoniae*	0	0	NA	NA	0	0	NA	NA	0.5	0	NA	0.499	1.0	0.5	0.51 (0.009–2.78)	0.686	2.1	2.2	1.04 (0.40–2.75)	1
*Staphylococcus aureus*	1.0	0.7	0.77 (0.17–3.40)	1	6.8	10.6	1.56 (0.98–2.48)	0.075	5.2	12.7	2.46 (1.49–4.06)	<0.001	3.4	15.3	4.49 (2.50–8.06)	<0.001	4.5	16.7	3.68 (2.19–6.18)	<0.001
**Any bacteria**	1.0	0.7	0.77 (0.17–3.40)	1	7.3	10.6	1.45 (0.92–2.28)	0.132	6.2	13.0	2.09 (1.31–3.34)	0.002	4.5	16.1	3.61 (2.15–6.08)	<0.001	6.7	19.2	2.88 (1.86–4.44)	<0.001

**NPS mother**	*n* = 415	*n* = 414			*n* = 402	*n* = 400			*n* = 397	*n* = 395			*n* = 390	*n* = 390			*n* = 386	*n* = 381		
GBS	0	0	NA	NA	0	0	NA	NA	0	0	NA	NA	0	0	NA	NA	0..3	0.3	1.01 (0.06–16.1)	1
*S. pneumoniae*	0	1.4	NA	NA	0.5	0.8	1.51 (0.25–8.97)	0.686	1.0	0.8	0.75 (0.17–3.35)	1	0.8	1.8	2.33 (0.61–8.96)	0.341	0.3	1.8	7.09 (0.88–57.4)	0.037
*S. aureus*	2.7	1.4	0.55 (0.20–1.46)	0.327	1.7	4.0	2.30 (0.96–8.97)	0.686	3.5	5.6	1.58 (0.82–3.04)	0.177	3.1	9.2	3.00 (1.85–5.68)	<0.001	2.8	12.6	4.42 (2.33–8.38)	<0.001
**Any bacteria**	2.7	2.9	1.09 (0.49–2.45)	0.836	2.2	4.8	2.12 (0.97–4.63)	0.057	4.3	6.3	1.48 (0.81–2.69)	0.209	3.8	10.5	2.73 (1.54–4.86)	<0.001	3.4	14.7	4.36 (2.43–7.85)	<0.001

**Breast milk**	*n* = 0	*n* = 0			*n* = 399	*n* = 394			*n* = 397	*n* = 385			*n* = 387	*n* = 388			*n* = 384	*n* = 381		
GBS	—	—	—	—	0.5	0.3	0.51 (0.05–5.56)	1	0.5	0.3	0.50 (0.05–5.52)	1	0.3	0.3	1.00 (0.006–15.9)	1	0	0	NA	NA
*S. pneumoniae*	—	—	—	—	0	0	NA	NA	0	0	NA	NA	0	0.0	NA	NA	0	0	NA	NA
*S. aureus*	—	—	—	—	1.8	4.8	2.75 (1.17–6.47)	0.017	2.0	5.1	2.51 (1.12–5.64)	0.021	0.8	5.7	7.31 (2.21–24.2)	<0.001	1.3	3.7	2.82 (1.03–7.76)	0.038
**Any bacteria**	—	—	—	—	2.3	5.1	2.25 (1.04–4.88)	0.038	2.5	5.3	2.11 (1.01–4.42)	0.045	1.0	5.9	5.74 (2.00–16.4)	<0.001	1.3	3.7	2.82 (1.03–7.76)	0.038

**Vaginal swabs**	*n* = 415	*n* = 414			*n* = 0	*n* = 0			*n* = 396	*n* = 386			*n* = 0	*n* = 0			*n* = 0	*n* = 0		
GBS	0.5	0.2	0.50 (0.05–5.51)	1	—	—	—	—	0.3	1.5	6.06 (0.73–50.1)	0.068	—	—	—	—	—	—	—	—
*S. pneumoniae*	0	0	NA	NA	—	—	—	—	0	0	NA	NA	—	—	—	—	—	—	—	—
*S.aureus*	1.7	0	NA	NA	—	—	—	—	1.0	6.9	6.82 (2.41–19.3)	<0.001	—	—	—	—	—	—	—	—
**Any bacteria**	2.2	0.2	0.11 (0.01–0.88)	0.021	—	—	—	—	1.3	8.4	6.67 (2.63–16.9)	<0.001	—	—	—	—	—	—	—	—

Abbreviations: AZI, azithromycin; GBS, group B streptococci; NPS, nasopharyngeal swab; P'bo– placebo; PR, Prevalence Ratio.

Azithromycin resistance was determined by E-test following the CLSI 2014 guidelines for the performance of test and interpretation of results:

(i) *S. aureus* isolates with E-test values ≥8 μg/mL

(ii) *S. pneumoniae*/GBS isolates with E-test values ≥2 μg/mL.l

a At day 0, nasopharyngeal and vaginal swabs in mothers were collected before the intervention and nasopharyngeal swabs in newborns after the intervention.

b Vaginal swab was collected at day 8–10 instead.

## Appendix A Supporting information

The following supplementary materials are available for this article:

**Table S1**. Bacterial carriage in newborns and mothers among participants treated more than 2 h before delivery.

**Table S2**. Bacterial carriage in newborns and mothers *per protocol* analysis.

**Table S3**. Acquisition of carriage in newborns and mothers. Intention-to-treat analysis.

## References

[1] Seale A.C., Mwaniki M., Newton C.R., Berkley J.A. (2009). Maternal and early onset neonatal bacterial sepsis: burden and strategies for prevention in sub-Saharan Africa. Lancet Infect Dis.

[2] Cutland C.L., Madhi S.A., Zell E.R., Kuwanda L., Laque M., Groome M. (2009). Chlorhexidine maternal-vaginal and neonate body wipes in sepsis and vertical transmission of pathogenic bacteria in South Africa: a randomised, controlled trial. Lancet.

[3] Waters D., Jawad I., Ahmad A., Luksic I., Nair H., Zgaga L. (2011). Aetiology of community-acquired neonatal sepsis in low and middle income countries. J Glob Health.

[4] Zaidi A.K., Thaver D., Ali S.A., Khan T.A. (2009). Pathogens associated with sepsis in newborns and young infants in developing countries. Pediatr Infect Dis J.

[5] Chatzakis E., Scoulica E., Papageorgiou N., Maraki S., Samonis G., Galanakis E. (2011). Infant colonization by Staphylococcus aureus: role of maternal carriage. Eur J Clin Microbiol Infect Dis.

[6] Rudan I., Theodoratou E., Nair H., Marusic A., Campbell H. (2011). Reducing the burden of maternal and neonatal infections in low income settings. J Glob Health.

[7] Suara R.O., Adegbola R.A., Baker C.J., Secka O., Mulholland E.K., Greenwood B.M. (1994). Carriage of group B Streptococci in pregnant Gambian mothers and their infants. J Infect Dis.

[8] Drew R.H., Gallis H.A. (1992). Azithromycin–spectrum of activity, pharmacokinetics, and clinical applications. Pharmacotherapy.

[9] Nosten F., McGready R., D'Alessandro U., Bonell A., Verhoeff F., Menendez C. (2006). Antimalarial drugs in pregnancy: a review. Curr Drug Saf.

[10] Orton L.C., Omari A.A. (2008). Drugs for treating uncomplicated malaria in pregnant women. Cochrane Database Syst Rev.

[11] Fry A.M., Jha H.C., Lietman T.M., Chaudhary J.S., Bhatta R.C., Elliott J. (2002). Adverse and beneficial secondary effects of mass treatment with azithromycin to eliminate blindness due to trachoma in Nepal. Clin Infect Dis.

[12] Leach A.J., Shelby-James T.M., Mayo M., Gratten M., Laming A.C., Currie B.J. (1997). A prospective study of the impact of community-based azithromycin treatment of trachoma on carriage and resistance of *Streptococcus pneumoniae*. Clin Infect Dis.

[13] Harding-Esch E.M., Edwards T., Mkocha H., Munoz B., Holland M.J., Burr S.E. (2010). Trachoma prevalence and associated risk factors in the gambia and Tanzania: baseline results of a cluster randomised controlled trial. PLoS Negl Trop Dis.

[14] Burr S.E., Milne S., Jafali J., Bojang E., Rajasekhar M., Hart J. (2014). Mass administration of azithromycin and *Streptococcus pneumoniae* carriage: cross-sectional surveys in the Gambia. Bull World Health Organ.

[15] Porco T.C., Gebre T., Ayele B., House J., Keenan J., Zhou Z. (2009). Effect of mass distribution of azithromycin for trachoma control on overall mortality in Ethiopian children: a randomized trial. JAMA.

[16] van den Broek N.R., White S.A., Goodall M., Ntonya C., Kayira E., Kafulafula G. (2009). The APPLe study: a randomized, community-based, placebo-controlled trial of azithromycin for the prevention of preterm birth, with meta-analysis. PLoS Med.

[17] Unger H.W., Ome-Kaius M., Wangnapi R.A., Umbers A.J., Hanieh S., Suen C.S. (2015). Sulphadoxine-pyrimethamine plus azithromycin for the prevention of low birthweight in Papua New Guinea: a randomised controlled trial. BMC Med.

[18] Di Renzo G.C., Melin P., Berardi A., Blennow M., Carbonell-Estrany X., Donzelli G.P. (2015). Intrapartum GBS screening and antibiotic prophylaxis: a European consensus conference. J Matern Fetal Neonatal Med.

[19] Schrag S.J., Verani J.R. (2013). Intrapartum antibiotic prophylaxis for the prevention of perinatal group B streptococcal disease: experience in the United States and implications for a potential group B streptococcal vaccine. Vaccine.

[20] Sigauque B., Roca A., Mandomando I., Morais L., Quinto L., Sacarlal J. (2009). Community-acquired bacteremia among children admitted to a rural hospital in Mozambique. Pediatr Infect Dis J.

[21] Roca A., Oluwalana C., Camara B., Bojang A., Burr S., Davis T.M. (2015). Prevention of bacterial infections in the newborn by pre-delivery administration of azithromycin: study protocol of a randomized efficacy trial. BMC Pregnancy Childbirth.

[22] Jasseh M., Webb E.L., Jaffar S., Howie S., Townend J., Smith P.G. (2011). Reaching millennium development goal 4-the Gambia. Trop Med Int Health.

[23] Bottomley C., Bojang A., Smith P.G., Darboe O., Antonio M., Foster-Nyarko E. (2015). The impact of childhood vaccines on bacterial carriage in the nasopharynx: a longitudinal study. Emerg Themes Epidemiol.

[24] Salman S., Davis T.M., Page-Sharp M., Camara B., Oluwalana C., Bojang A. (2015 Dec 28). Pharmacokinetics of transfer of azithromycin into the breast milk of African mothers. Antimicrob Agents Chemother.

[25] Unger H.W., Aho C., Ome-Kaius M., Wangnapi R.A., Umbers A.J., Jack W. (2015 Feb 11). Impact of intermittent preventive treatment in pregnancy with azithromycin-containing regimens on maternal nasopharyngeal carriage and antibiotic sensitivity of *Streptococcus pneumoniae, Haemophilus influenzae* and *Staphylococcus aureus*—a cross-sectional survey at delivery. J Clin Microbiol.

[26] Revised guidelines for prevention of early-onset group B streptococcal (GBS) infection (1997). American Academy of Pediatrics Committee on Infectious Diseases and Committee on Fetus and Newborn. Pediatrics.

[27] Andersson D.I., Hughes D. (2010). Antibiotic resistance and its cost: is it possible to reverse resistance?. Nat Rev Microbiol.

