# Supplementary material for: Oral azithromycin given during labour decreases bacterial carriage in the mothers and their offspring: a double-blind randomized trial
Source: Clin Microbiol Infect. 2016 Jun;22(6):565.e1–9. doi: 10.1016/j.cmi.2016.03.005 (PMC4936760; doi:10.1016/j.cmi.2016.03.005)
Supplement: Supplementary file 1 [file mmc1.docx]

**Web-Table 1:**  Bacterial colonization in newborns and mothers among participants treated more than 2 hours before delivery.

|  | **Day 0^1^** | | | | **Day 3** | | | | **Day 6^2^** | | | | **Day 14** | | | | **Day 28** | | | |
| --- | --- | --- | --- | --- | --- | --- | --- | --- | --- | --- | --- | --- | --- | --- | --- | --- | --- | --- | --- | --- |
|  | P’bo (%) | AZI (%) | PR (95%CI) | p-value | P’bo (%) | AZI (%) | PR (95%CI) | p-value | P’bo (%) | AZI (%) | PR (95%CI) | p-value | P’bo (%) | AZI (%) | PR (95%CI) | p-value | P’bo (%) | AZI (%) | PR (95%CI) | p-value |
| **NPS newborn** | N=263 | N=250 |  |  | N=244 | N=233 |  |  | N=236 | N=226 |  |  | N=235 | N=223 |  |  | N=229 | N=214 |  |  |
| GBS | 2.3 | 0.8 | 0.35 (0.07,1.72) | 0.286 | 9.4 | 0.4 | 0.05 (0.01,0.33) | <0.001 | 4.7 | 0.9 | 0.19 (0.04,0.85) | 0.021 | 6.0 | 0 | NA | <0.001 | 2.2 | 0 | NA | 0.062 |
| *S. pneumoniae* | 0 | 0.4 | NA | NA | 0.8 | 0 | NA | NA | 4.7 | 0.4 | 0.09 (0.01,0.73) | 0.006 | 16.6 | 7.2 | 0.43 (0.25,0.75) | 0.002 | 32.8 | 20.6 | 0.63 (0.45,0.87) | 0.005 |
| *S. aureus* | 4.2 | 1.6 | 0.38 (0.12,1.19) | 0.115 | 59.0 | 20.6 | 0.35 (0.27,0.46) | <0.001 | 62.3 | 23.0 | 0.37 (0.29,0.48) | <0.001 | 45.1 | 18.4 | 0.41 (0.30,0.56) | <0.001 | 35.4 | 25.2 | 0.71 (0.53,0.95) | 0.023 |
| **Any bacteria** | 6.5 | 2.4 | 0.37 (0.15,0.93) | 0.032 | 63.5 | 20.6 | 0.32 (0.25,0.42) | <0.001 | 63.6 | 23.5 | 0.37 (0.29,0.48) | <0.001 | 57.4 | 23.8 | 0.41 (0.32,0.54) | <0.001 | 55.9 | 42.1 | 0.75 (0.62,0.91) | 0.004 |
| **NPS mother** | N=266 | N=258 |  |  | N=254 | N=245 |  |  | N=250 | N=241 |  |  | N=247 | N=237 |  |  | N=242 | N=232 |  |  |
| GBS | 0.4 | 1.2 | 3.09 (0.32,29.5) | 0.366 | 0 | 0 | NA | NA | 0 | 0 | NA | NA | 0 | 0 | NA | NA | 0 | 0 | NA | NA |
| *S. pneumoniae* | 18.0 | 14.0 | 0.77 (0.52,1.15) | 0.200 | 17.3 | 0.8 | 0.05 (0.01,1.19) | <0.001 | 16.8 | 1.7 | 0.10 (0.04,0.27) | <0.001 | 21.1 | 4.6 | 0.22 (0.12,0.41) | <0.001 | 24.4 | 4.3 | 0.18 (0.09,0.34) | <0.001 |
| *S. aureus* | 17.7 | 19.0 | 1.07 (0.75,1.54) | 0.735 | 25.6 | 8.6 | 0.33 (0.21,0.53) | <0.001 | 26.4 | 10.4 | 0.39 (0.26,0.60) | <0.001 | 25.9 | 4.6 | 0.46 (0.30,0.68) | <0.001 | 26.4 | 17.2 | 0.65 (0.46,0.93) | <0.001 |
| **Any bacteria** | 33.8 | 31.4 | 0.93 (0.73,1.19) | 0.577 | 40.9 | 9.4 | 0.23 (0.15,0.35) | <0.001 | 39.6 | 12.0 | 0.30 (0.21,0.44) | <0.001 | 42.5 | 15.2 | 0.36 (0.26,0.50) | <0.001 | 45.5 | 21.1 | 0.46 (0.35,0.62) | <0.001 |
| **Breast Milk** | N=0 | N=0 |  |  | N=251 | N=239 |  |  | N=250 | N=241 |  |  | N=244 | N=236 |  |  | N=240 | N=232 |  |  |
| GBS | - | - | - | - | 8.4 | 1.3 | 0.15 (0.05,0.50) | <0.001 | 8.8 | 2.1 | 0.24 (0.09,0.61) | 0.001 | 7.0 | 0.4 | 0.06 (0.01,0.45) | <0.001 | 1.3 | 0.0 | NA | 0.200 |
| *S. pneumoniae* | - | - | - | - | 0.8 | 0 | NA | NA | 0 | 0 | NA | NA | 0.8 | 0.0 | NA | 0.499 | 0.0 | 0.0 | NA | NA |
| *S. aureus* | - | - | - | - | 15.1 | 9.2 | 0.61 (0.37,1.00) | <0.001 | 14.0 | 10.0 | 0.71 (0.44,1.16) | 0.211 | 11.5 | 8.5 | 0.74 (0.43,1.27) | 0.290 | 9.2 | 6.0 | 0.66 (0.35,1.26) | 0.200 |
| **Any bacteria** | - | - | - | - | 22.7 | 10.5 | 0.46 (0.30,0.71) | <0.001 | 20.4 | 11.6 | 0.57 (0.37,0.87) | 0.010 | 16.4 | 8.9 | 0.54 (0.33,0.89) | 0.019 | 10.0 | 6.0 | 0.60 (0.32,1.14) | 0.100 |
| **Vaginal swabs** | N=266 | N=258 |  |  | N=0 | N=0 |  |  | N=251 | N=239 |  |  | N=0 | N=0 |  |  | N=0 | N=0 |  |  |
| GBS | 17.7 | 20.9 | 1.18 (0.83,1.68) | 0.376 | - | - | - | - | 13.1 | 3.3 | 0.25 (0.12,0.54) | <0.001 | - | - | - | - | - | - | - | - |
| *S. pneumoniae* | 0 | 0 | NA | NA | - | - | - | - | 0 | 0 | NA | NA | - | - | - | - | - | - | - | - |
| *S. aureus* | 13.5 | 12.4 | 0.92 (0.59,1.43) | 0.795 | - | - | - | - | 14.7 | 10.0 | 0.68 (0.42,1.10) | 0.133 | - | - | - | - | - | - | - | - |
| **Any bacteria** | 27.8 | 27.9 | 1.00 (0.76,1.32) | 1.000 | - | - | - | - | 24.3 | 13.0 | 0.53 (0.36,0.79) | 0.002 | - | - | - | - | - | - | - | - |

^1^At day 0, NPS and VS in mothers was collected before the intervention and in newborns after the intervention.

^2^ VS was collected at day 8-10 instead

P’bo=Placebo; AZI=Azithromycin; PR=Prevalence Ratio; CI=Confidence Interval; GBS=Group B *Streptococcus*

**Web-table 2:** Bacterial colonization in newborns and mothers *per-protocol* analysis.

|  | **Day 0^1^** | | | | **Day 3** | | | | **Day 6^2^** | | | | **Day 14** | | | | **Day 28** | | | |
| --- | --- | --- | --- | --- | --- | --- | --- | --- | --- | --- | --- | --- | --- | --- | --- | --- | --- | --- | --- | --- |
|  | P’bo (%) | AZI (%) | PR (95%CI) | p-value | P’bo (%) | AZI (%) | PR (95%CI) | p-value | P’bo (%) | AZI (%) | PR (95%CI) | p-value | P’bo (%) | AZI (%) | PR (95%CI) | p-value | P’bo (%) | AZI (%) | PR (95%CI) | p-value |
| **NPS newborn** | N=405 | N=390 |  |  | N=388 | N=370 |  |  | N=379 | N=359 |  |  | N=372 | N=353 |  |  | N=364 | N=346 |  |  |
| GBS | 1.5 | 0.8 | 0.52 (0.13,2.06) | 0.506 | 10.1 | 0.8 | 0.08 (0.03,0.26) | <0.001 | 5.3 | 1.1 | 0.21 (0.07,0.61) | 0.001 | 4.8 | 0.8 | 0.18 (0.05,0.59) | 0.001 | 3.3 | 0.6 | 0.18 (0.04,0.78) | 0.012 |
| *S. pneumoniae* | 0.2 | 0.3 | 1.04 (0.07,16.5) | 1.000 | 1.3 | 0.8 | 0.63 (0.15,2.61) | 0.726 | 4.2 | 0.8 | 0.20 (0.06,0.67) | 0.004 | 19.9 | 8.2 | 0.41 (0.28,0.62) | <0.001 | 36.8 | 24.6 | 0.67 (0.53,0.84) | <0.001 |
| *S. aureus* | 3.7 | 1.5 | 0.42 (0.16,1.06) | 0.076 | 57.5 | 24.9 | 0.43 (0.36,0.53) | <0.001 | 63.3 | 27.6 | 0.44 (0.36,0.52) | <0.001 | 43.0 | 21.5 | 0.50 (0.40,0.63) | <0.001 | 34.1 | 25.4 | 0.75 (0.59,0.94) | 0.014 |
| **Any bacteria** | 5.4 | 2.3 | 0.42 (0.20,0.91) | 0.027 | 62.1 | 25.7 | 0.41 (0.34,0.50) | <0.001 | 65.2 | 28.4 | 0.44 (0.36,0.52) | <0.001 | 57.0 | 28.0 | 0.49 (0.41,0.59) | <0.001 | 59.6 | 45.4 | 0.76 (0.66,0.88) | <0.001 |
| **NPS mother** | N=407 | N=392 |  |  | N=395 | N=384 |  |  | N=388 | N=377 |  |  | N=381 | N=369 |  |  | N=375 | N=367 |  |  |
| GBS | 0.5 | 0.8 | 1.56 (0.26,9.27) | 0.681 | 0.3 | 0 | NA | 1.000 | 0 | 0 | NA | NA | 0 | 0 | NA | NA | 0.5 | 0.3 | 0.51 (0.05,5.61) | 1.000 |
| *S. pneumoniae* | 17.2 | 16.3 | 0.95 (0.70,1.29) | 0.777 | 18.0 | 1.0 | 0.06 (0.02,0.16) | <0.001 | 16.2 | 1.3 | 0.08 (0.03,0.20) | <0.001 | 22.0 | 3.0 | 0.22 (0.12,0.41) | <0.001 | 23.5 | 4.4 | 0.19 (0.11,0.31) | <0.001 |
| *S. aureus* | 18.9 | 18.9 | 1.00 (0.75,1.33) | 1.000 | 25.1 | 7.6 | 0.30 (0.20,0.44) | <0.001 | 26.8 | 7.7 | 0.29 (0.20,0.42) | <0.001 | 26.0 | 9.2 | 0.46 (0.30,0.68) | <0.001 | 26.9 | 14.7 | 0.55 (0.41,0.74) | <0.001 |
| **Any bacteria** | 34.4 | 33.2 | 0.96 (0.79,1.17) | 0.765 | 39.7 | 8.6 | 0.22 (0.15,0.31) | <0.001 | 39.9 | 9.0 | 0.23 (0.16,0.32) | <0.001 | 43.8 | 11.7 | 0.27 (0.20,0.36) | <0.001 | 45.1 | 19.1 | 0.42 (0.33,0.54) | <0.001 |
| **Breast Milk** | N=0 | N=0 |  |  | N=393 | N=378 |  |  | N=391 | N=378 |  |  | N=380 | N=373 |  |  | N=373 | N=368 |  |  |
| GBS | - | - | - | - | 6.6 | 0.8 | 0.12 (0.04,0.39) | <0.001 | 7.7 | 1.9 | 0.24 (0.11,0.54) | <0.001 | 6.6 | 0.8 | 0.12 (0.04,0.40) | <0.001 | 1.6 | 0.0 | NA | 0.200 |
| *S. pneumoniae* | - | - | - | - | 0.5 | 0 | NA | 0.500 | 0 | 0 | NA | NA | 0.8 | 0.0 | NA | 0.499 | 0.0 | 0.0 | NA | NA |
| *S. aureus* | - | - | - | - | 15.8 | 7.4 | 0.48 (0.31,0.72) | <0.001 | 16.6 | 8.2 | 0.49 (0.33,0.74) | <0.001 | 12.6 | 7.8 | 0.62 (0.40,0.95) | 0.030 | 10.7 | 5.2 | 0.48 (0.28,0.82) | 0.006 |
| **Any bacteria** | - | - | - | - | 21.6 | 8.2 | 0.38 (0.26,0.56) | <0.001 | 22.3 | 9.8 | 0.44 (0.31,0.63) | <0.001 | 17.6 | 8.6 | 0.49 (0.33,0.72) | <0.001 | 12.1 | 5.2 | 0.43 (0.26,0.72) | 0.001 |
| **Vaginal swabs** | N=408 | N=392 |  |  | N=0 | N=0 |  |  | N=391 | N=379 |  |  | N=0 | N=0 |  |  | N=0 | N=0 |  |  |
| GBS | 15.9 | 18.2 | 1.17 (0.86,1.58) | 0.349 | - | - | - | - | 13.3 | 4.2 | 0.32 (0.18,0.55) | <0.001 | - | - | - | - | - | - | - | - |
| *S. pneumoniae* | 0 | 0 | NA | NA | - | - | - | - | 0 | 0 | NA | NA | - | - | - | - | - | - | - | - |
| *S. aureus* | 16.4 | 12.5 | 0.76 (0.54,1.07) | 0.132 | - | - | - | - | 14.1 | 9.0 | 0.64 (0.43,0.95) | 0.032 | - | - | - | - | - | - | - | - |
| **Any bacteria** | 28.9 | 25.8 | 0.89 (0.71,1.12) | 0.341 | - | - | - | - | 24.0 | 12.9 | 0.54 (0.39,0.74) | <0.001 | - | - | - | - | - | - | - | - |

^1^At day 0, NPS in mothers was collected before the intervention and in newborns after the intervention.

^2^ VS was collected at day 8-10 instead

P’bo=Placebo; AZI=Azithromycin; PR=Prevalence Ratio; CI=Confidence Interval; GBS=Group B *Streptococcus*

**Web-Table 3**: Acquisition of colonization in newborns and mothers. Intention to treat analysis.

|  |  | **Acquisition days 0-6** | | | | | **Acquisition days 7-28** | | | | |
| --- | --- | --- | --- | --- | --- | --- | --- | --- | --- | --- | --- |
|  |  | P’bo (%) | | AZI (%) | PR (95%CI) | p-value | P’bo (%) | AZI (%) | PR (95%CI) | | p-value |
| **NPS newborn^1^** |  | | | | | | | | | | |
|  | GBS | | 13.0 | 2.1 | 0.16 (0.08,0.34) | <0.001 | 5.5 | 0.9 | 0.16 (0.05,0.52) | <0.001 | |
|  | *S. pneumoniae* | | 4.4 | 1.6 | 0.36 (0.14,0.90) | 0.032 | 38.7 | 26.5 | 0.68 (0.55,0.85) | 0.001 | |
|  | *S. aureus* | | 71.5 | 34.1 | 0.48 (0.41,0.56) | <0.001 | 40.4 | 18.1 | 0.45 (0.32,0.62) | <0.001 | |
|  | **Any bacteria** | | 75.1 | 36.0 | 0.48 (0.41,0.55) | <0.001 | 66.2 | 39.8 | 0.60 (0.50,0.73) | <0.001 | |
| **NPS mother^1^** |  | | | | | | | | | | |
|  | GBS | | 0.8 | 0.5 | 0.67 (0.11,3.97) | 1.000 | 0.5 | 0.3 | 0.50 (0.05,5.52) | 1.000 | |
|  | *S. pneumoniae* | | 28.9 | 16.5 | 0.57 (0.43,0.75) | <0.001 | 27.8 | 5.3 | 0.19 (0.12,0.31) | <0.001 | |
|  | *S. aureus* | | 39.8 | 27.2 | 0.68 (0.56,0.83) | <0.001 | 26.8 | 15.5 | 0.58 (0.42,0.79) | 0.001 | |
|  | **Any bacteria** | | 58.9 | 40.1 | 0.38 (0.59,0.79) | <0.001 | 50.9 | 20.9 | 0.41 (0.32,0.52) | <0.001 | |
| **Breast Milk^2^** |  | | | | | | | | | | |
|  | GBS | | 11.9 | 2.0 | 0.17 (0.08,0.36) | <0.001 | 5.4 | 0.8 | 0.15 (0.04,0.50) | <0.001 | |
|  | *S. pneumoniae* | | 0.5 | 0 | NA | 0.499 | 0.8 | 0 | NA | 0.249 | |
|  | *S. aureus* | | 23.9 | 12.2 | 0.51 (0.37,0.71) | <0.001 | 16.8 | 9.0 | 0.54 (0..35,0.81) | 0.003 | |
|  | **Any bacteria** | | 32.2 | 14.0 | 0.44 (0.33,0.58) | <0.001 | 20.5 | 9.7 | 0.47 (0.32,0.70) | <0.001 | |

*^1^At day 0, NPS in mothers was collected before the intervention and in newborns after the intervention.*

*^2^ First breast milk sample collected at day 3.*

P’bo=Placebo; AZI=Azithromycin; PR=Prevalence Ratio; CI=Confidence Interval; GBS=Group B *Streptococcus*
